# Bio-Based Materials for Packaging

**DOI:** 10.3390/ijms23073611

**Published:** 2022-03-25

**Authors:** Raffaele Porta, Mohammed Sabbah, Prospero Di Pierro

**Affiliations:** 1 Department of Chemical Sciences, University of Naples Federico II, 80126 Naples, Italy; 2 Department of Nutrition and Food Technology, An-Najah National University, Nablus P400, Palestine; m.t.m.sabbah@gmail.com; 3 Department of Agriculture, University of Naples Federico II, Portici, 80055 Naples, Italy; dipierro@unina.it

Plastic pollution is currently one of the most pressing environmental problems, especially in countries with a low recycling rate that is mainly due to the insufficient collection of plastic waste. Plastic production increased from 2.3 million tons in 1950 to about 450 million tons by 2015 and is expected to double by 2050 [1]. Most conventional plastic materials are manufactured with fossil-based polymers and are able to decompose spontaneously for a very long time in response to natural stimuli, both physical and chemical, that generate secondary micro- and nano-plastic pollution [2,3,4]. Since they are non-biodegradable, being an uncommon target for bacteria, the accumulation of plastic objects and particles in the Earth’s environment adversely affects humans, wildlife, and their habitat [5,6,7]. The increased awareness of environmental protection has stimulated the development of different bio-based polymers, also derived from renewable sources, as matrix alternatives to petroleum-derived polymers to produce innovative “bioplastics” for industrial exploitation (Figure 1) [1,8]. The main physicochemical and functional properties of bio-based polymers, generally investigated for their application as components of packaging materials, are listed in Figure 2. It is important to underline that not all bio-based plastics are biodegradable, and not all biodegradable plastics are bio-based. Examples are BioPET, the polyethylene terephthalate (PET) produced from biomass, that is not biodegradable, as well as some fossil-derived materials (such as those made with polycaprolactone) that are biodegradable [9].

Nowadays, most of the materials used in the packaging industries derive from fossil fuels and, mainly when exploited for short-term storage packaging, these materials represent a serious environmental concern. Statistics on packaging waste in the 27 EU member States and some non-member countries have been recently disclosed [10]. In particular, the reported data indicate that packaging waste generated in 2019 was estimated at 177.4 kg per inhabitant and that plastics was about 20% of the total packaging waste, rising by 13.3 million tonnes from 2009 to 2019 (+20.1%). Over the 10-year period, plastic packaging material was the second most significant packaging waste generated after paper and cardboard. On the other hand, the 2019 recycling rates of all packaging wastes and only plastic waste, generated by the EU Member States plus EEA/EFTA countries, were 65 and 40%, respectively, where the recycling rate of plastic packaging waste exclusively referred to material that is recycled back into plastic. It is important to outline that the amount of plastic material specifically used for food packaging is very high (approximately two-thirds of all the material produced) because synthetic packaging is effective in preventing the transfer of water, gas, and flavorings between the wrapped or coated food and the surrounding medium. Thus, the vast majority of food products, especially processed foods, are currently wrapped, and new, more effective types of packaging are constantly being developed. In fact, food is increasingly sold in grocery stores in plastic containers, often separated by multiple layers of plastic, then placed into plastic bags that sometimes, especially for takeout food, also contain plastic cutlery, napkins, and straws. Therefore, as the main consumer of single-use packaging, the food sector is one of the biggest responsible for plastic pollution, and the transition to sustainable packaging in this sector still remains slow and limited [11]. In conclusion, radical changes and innovations in the production system are urgently needed to reverse this polluting process by also strengthening producer responsibility.

Numerous bio-based and biodegradable polymers, as well as edible polymers, have been studied and proposed in the last twenty years for the production of food packaging materials—as shown by the number of research articles, reviews, book chapters reported in the scientific literature—in order to replace those produced from fossil-fuel-based polymers [12,13,14,15]. Several of these exhibit sufficient mechanical properties and satisfying barrier behaviors to control the transfer of moisture, oxygen, carbon dioxide, flavors, or other hydrophobic and volatile compounds in order to prevent quality deterioration of different food products and extend their shelf life.

Some of these innovative materials have also been shown to be able to maintain or enhance the safety and sensory quality of the product by adding beneficial functional activities to packaged foods when applied on their surface or inside between different layers of food. Therefore, the search for suitable protective packaging derived from renewable sources is becoming an attractive topic, especially for the food industry, which is interested in maintaining the quality and freshness of products during the time necessary for their marketing and consumption and decreasing plastic pollution. Along with the efforts pursued to improve food quality, worldwide attention is thus focused on reducing plastic waste to counteract environmental degradation. This also explains the growing interest in the exploitation of agricultural waste as a feedstock for the production of sustainable packaging, as well as the encouraged exploration of bio-based renewable materials [16,17]. 

However, unfortunately, the use of bio-based materials for food packaging has been limited so far by their poor barrier properties and weak mechanical properties [18]. It is generally recognized by researchers that replacing food plastic packaging with one manufactured with only one full stand-alone biopolymer is a tough goal to reach. For this reason, natural polymers are frequently blended with other polymers, added with reinforcing nanoparticles, or preliminarily modified either chemically or enzymatically in order to extend their applications. Thus, research and development of new bionanocomposite materials for food packaging are expected to grow in the near future with the aim to reduce plastic’s environmental impact [19].

In the current Special Issue of the *International Journal of Molecular Sciences,* we edited several papers highlighting additives or processing aids able to improve the physicochemical and functional properties of different bio-based materials. In the article entitled “Novel Bio-Based Materials and Applications in Antimicrobial Food Packaging: Recent Advances and Future Trends”, Tan et al. [20] presented an extensive overview of the development of various bio-based materials obtained from different sources, including polysaccharides, proteins, and lipids. The application of these materials in the production of antimicrobial food packaging containing clay and silicate-, biopolymer-, metal-, nanocellulose-, and layered double hydroxide-based bio-nanocomposites was also described. An additional research article by Contessa et al. [21], entitled “New Active Packaging Based on Biopolymeric Mixture Added with Bacteriocin as Active Compound”, described a chitosan/agar-agar bioplastic film incorporating bacteriocin extracted from *Lactobacillus sakei* as an antibacterial agent. When used for Minas Frescal cream cheese packaging, this material was shown to contribute to a significant increase in cheese microbiological stability, releasing the active compound into the food during its storage. Furthermore, two articles focused on the enrichment of a cycloolefin copolymer, as well as a protein-based film, with natural antioxidant agents. In the first case, Masek and Plota demonstrated in their article, “Influence of a Natural Plant Antioxidant on the Ageing Process of Ethylene-norbornene Copolymer (Topas)” [22], the stabilizer effect of hesperidin, a flavanone glycoside present in citrus-based fruits, on the aging behavior of the ethylene–norbornene copolymers. In the second article, entitled “Lignin/Carbohydrate Complex Isolated from *Posidonia oceanica* Sea Balls (Egagropili): Characterization and Antioxidant Reinforcement of Protein-Based Films”, Mirpoor et al. [23] isolated a lignin/carbohydrate complex, formed by a phenol polymeric chain covalently bound to hemicellulose fragments, from egagropili lignocellulosic material. Such soluble lignin fraction, possessing a remarkable and stable antioxidant activity, was found to be easily incorporated into a hemp-protein-based film and released from the latter over time. Finally, the last two papers offered insight into the improvement of the physicochemical properties of selected materials. The article published by Galus et al., entitled “Effects of Candelilla and Carnauba Wax Incorporation on the Functional Properties of Edible Sodium Caseinate Films” [24], evaluated the effects of wax incorporation on the functional properties of edible sodium caseinate films. These findings showed that films containing candelilla wax exhibited more regular lipid reorganization, which resulted in their better water vapor barrier efficacy and mechanical resistance in comparison to control films. In the other article, entitled “Low-Cost Biobased Coatings for AM60 Magnesium Alloys for Food Contact and Harsh Environment Applications”, Mangolini et al. [25] proposed a low-cost, stable, environmentally friendly, and safe bio-based polyamide coating as a promising candidate for food and beverage related applications in chemically aggressive environments.

All of these new studies clearly show the versatility, but also the complexity, of sustainable bio-based materials. We hope that the reported results can contribute to gaining a better understanding of how to further improve bio-based materials’ properties, increasing their potential as reliable alternatives to traditional synthetic plastics.

## Figures and Tables

**Figure 1:**
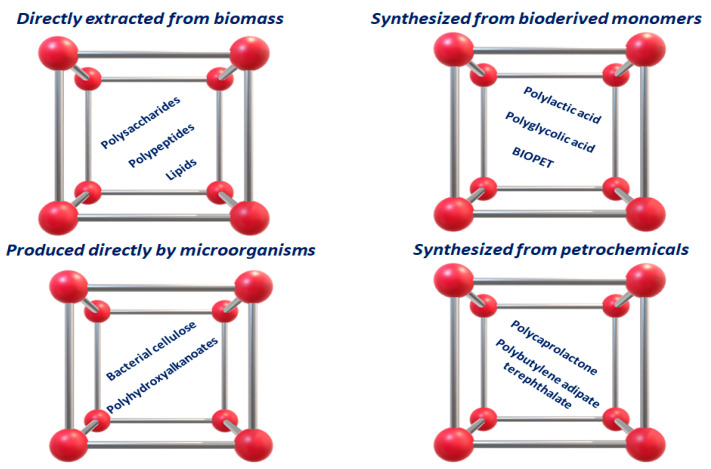
Main bio-based polymers investigated for packaging.

**Figure 2:**
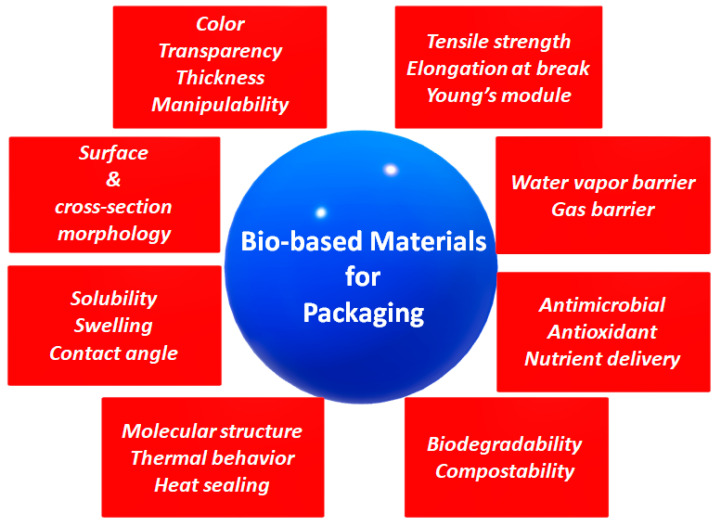
Main physicochemical and functional properties of bio-based polymers to be studied for their use as components of the packaging matrix.

## Data Availability

Not applicable.
